# The New Hampshire Journal of Medicine

**Published:** 1850-09

**Authors:** 


*The New Hampshire Journal of Medicine.* Edited by Edward H. Parker, A. M., M. D., August, 1850. Concord, New Hampshire.

We have received the first number of an able Medical Journal, the title of which we have given above. It is edited with ability, and we wish it an abundant and permanent prosperity. B.

